# DeepGhost: real-time computational ghost imaging via deep learning

**DOI:** 10.1038/s41598-020-68401-8

**Published:** 2020-07-09

**Authors:** Saad Rizvi, Jie Cao, Kaiyu Zhang, Qun Hao

**Affiliations:** School of Optics and Photonics, Beijing Institute of Technology, Key Laboratory of Biomimetic Robots and Systems, Ministry of Education, Beijing, 100081 China

**Keywords:** Imaging and sensing, Optical techniques

## Abstract

The potential of random pattern based computational ghost imaging (CGI) for real-time applications has been offset by its long image reconstruction time and inefficient reconstruction of complex diverse scenes. To overcome these problems, we propose a fast image reconstruction framework for CGI, called “DeepGhost”, using deep convolutional autoencoder network to achieve real-time imaging at very low sampling rates (10–20%). By transferring prior-knowledge from STL-10 dataset to physical-data driven network, the proposed framework can reconstruct complex unseen targets with high accuracy. The experimental results show that the proposed method outperforms existing deep learning and state-of-the-art compressed sensing methods used for ghost imaging under similar conditions. The proposed method employs deep architecture with fast computation, and tackles the shortcomings of existing schemes i.e., inappropriate architecture, training on limited data under controlled settings, and employing shallow network for fast computation.

## Introduction

Computational ghost imaging^1^ acquires spatial information about an unknown target by illuminating it with a series of random binary patterns generated by a spatial light modulator (SLM). For each projected pattern, the light intensity back-reflected from the target plane is recorded by an ordinary photodiode. By correlating intensity measurements with corresponding projected patterns, the target image is reconstructed. One downside of CGI is the requirement of a large number of measurements to produce a good-quality image, which increases its imaging time. Despite the emergence of basis scan schemes^2^, CGI (using random patterns) is still employed in many applications due to its simplicity, inherent encryption of patterns^3^, and ease of deployment^4^. Therefore, it is important to improve the efficiency of CGI by integrating it with some optimization technique to avoid complex (hardware based) methods^5^ that fail to reap the benefits of reduced cost and simplicity in ghost imaging (GI). Owing to its advantages of low cost, robustness against noise and scattering, and ability to operate over long spectral range, CGI is widely used in many applications^6–8^.

In order to make CGI practical, more specifically for real-time imaging, it is important to reduce its imaging time. The imaging time of CGI can be sub-categorized as data acquisition time and image reconstruction time. The data acquisition time of CGI depends on the required number of measurements and mainly on the projection rate of SLM. Recent advances in SLM technology make it easy to reduce data acquisition time by employing commercially available high-resolution digital micromirror devices (DMDs) operating at ~ 20 kHz. The acquisition time can also be reduced by employing some simple yet novel solutions^9,10^. Therefore, the image reconstruction time remains the main bottleneck towards achieving high speed imaging in CGI. This image reconstruction time can be reduced by employing an efficient image reconstruction framework.

Recently, compressive sensing (CS) techniques^11^ have been applied to recover an image with fewer (compressive) measurements. Although a promising technique, CS suffers from two inherent problems. First, to reconstruct an image from a few samples, CS algorithms require prior knowledge about the scene. However, for practical applications, images may not be sparse in a fixed basis, thereby limiting application flexibility. Second, the computational cost associated with most high-performance CS algorithms is very high, which increases reconstruction time, hence restricting their use in real-time applications. Although CS has been applied successfully in GI^12^, fast image reconstruction requires an alternative advanced method.

Recent years have seen the rise of Deep learning (DL) as a powerful technique for solving complex problems in computational imaging^13^. DL has the potential to significantly enhance the performance of GI for real-time applications. For some years, the GI community remained skeptic about using DL for fast image reconstruction, relying on basic correlation and probabilistic methods for target detection^14,15^. Recently, there have been some interesting studies that explore the potential of DL for GI^16–20^. For GI, the most relevant deep neural network model is the denoising autoencoder^21^. An autoencoder can be used as an unsupervised feature learner to extract features from high-dimensional data in a systematic fashion. For GI, the autoencoder model can be used to recover a clean image from an undersampled ghost image reconstructed from fewer measurements, thus reducing reconstruction time.

The existing DL methods applied to CGI have limited applicability due to: (a) inappropriate architecture, (b) training on limited data or targets, and (c) employing shallow network for real-time operation. These schemes can work under controlled settings but fail when tested on a large dataset with complex scenes and measurement noise. For example, in Ref.^16^ a stacked neural network model was used, confirming the potential of DL in CGI. The model employs a shallow fully connected network which is known to have computational complexity and is prone to data overfitting^22^. The model seems to work well with MNIST dataset, but its fully connected architecture is not suitable for complex image analysis. For image analysis, a more apt choice is the convolutional neural network (CNN)^23^. The work presented in Ref.^17^ proposed a better (autoencoder) model based on CNN for CGI. However, the network was only trained for a particular object with limited training dataset, therefore not utilizing the true power of CNN.

In this paper, we demonstrate a CGI system that employs deep convolutional autoencoder network (DCAN) to reconstruct real-time images, using only a photodiode and random binary patterns for target scanning. The proposed DCAN (called “DeepGhost”) strikes a balance between depth of layers and computation speed by employing a novel architecture for improved image recovery and fast network convergence. By employing innovations such as augmentation and transfer-learning, the proposed method can image complex unseen targets with high efficiency. Through simulations and experiments, we validate the superiority of our model by comparing it with existing DL^16,17^ and state-of-the-art compressive sensing algorithms^24^ used for GI under similar conditions.

## Results

### Simulations

**Figure 1:**
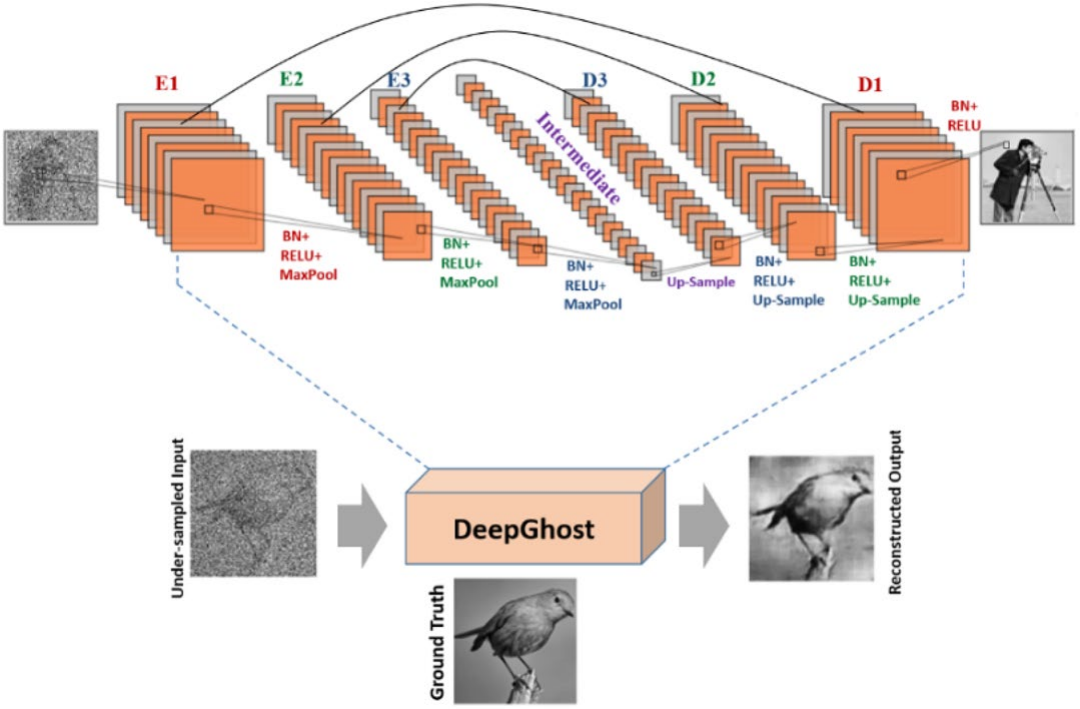
DeepGhost network architecture.

**Figure 2:**
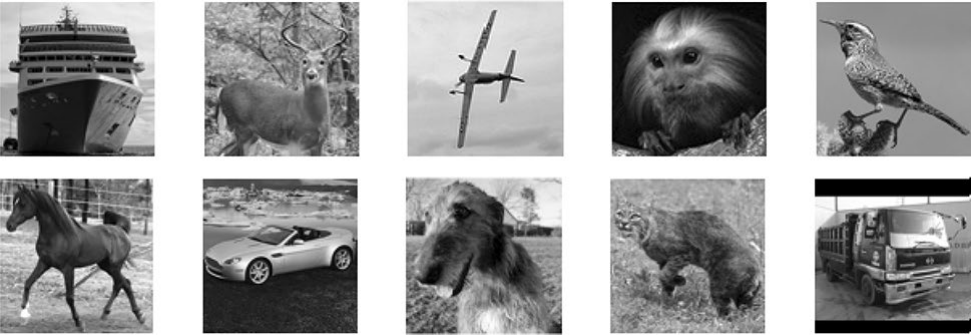
Sample images from 10 classes used for training.

The network architecture for DeepGhost is shown in Fig. 1. The idea is to feed the network with undersampled (10%, 15%, 2 0%, and 25%) target images (acquired from CGI setup) for clear target reconstruction. The proposed network is optimized for physical imaging setup by exhaustively testing through numerical simulations. For training and testing, STL-10^25^ dataset is used, which comprises of 10 classes: monkey, cat, dog, deer, car, truck, airplane, bird, horse, and ship. Sample image from each class is shown in Fig. 2.

### Comparison with conventional and CS algorithms

First, the performance of DeepGhost is evaluated through comparison with differential ghost imaging (DGI^26^) and compressive sensing methods^24^. The DeepGhost model is first trained on STL-10 data set (10,000 images), and then evaluated over a validation dataset (1,000 images) which is not seen during training. The same validation dataset is used as target images for DGI and CS based methods. In this paper, the sampling ratio ‘***S’*** is defined as the ratio between *Number* *of* *measurements* to *Image* *size* *in* *pixels*. For quantitative comparison, peak signal-to-noise ratio (PSNR) and Structural SIMilarity (SSIM)^27^ metrics are used.

### Results and analysis

**Figure 3:**
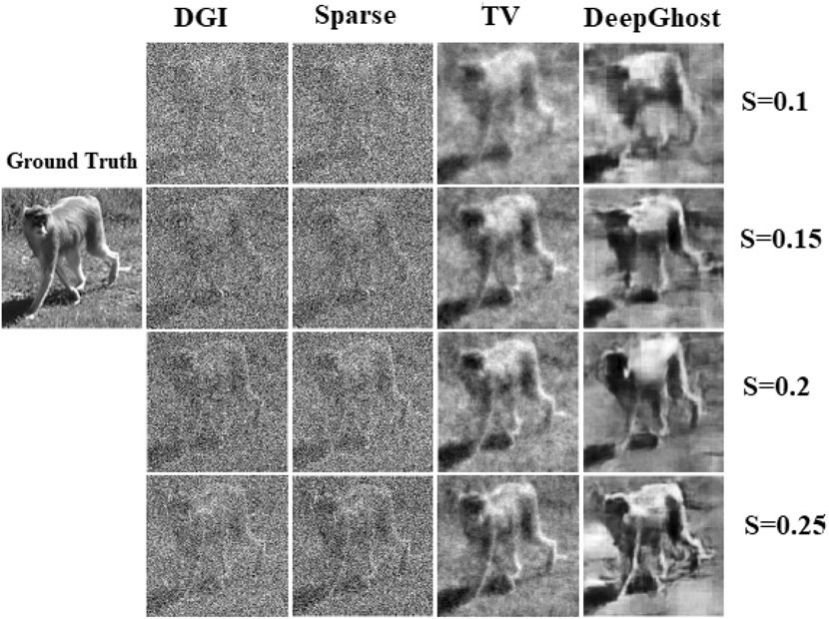
Qualitative comparison of reconstructions from different algorithms.

For qualitative comparison, an image from the “*monkey*” class of validation dataset is chosen. We evaluate the reconstruction results of DGI, Sparse, total variation (TV), and DeepGhost algorithms (see details in “Methods” ****section) for sampling ratios ranging from 0.1 to 0.25. We use Sparse and TV algorithms which are well-known high performance algorithms for specifically comparing the reconstruction quality. By visual inspection, it can be seen from Fig. 3 that the reconstruction results for TV and DeepGhost are almost identical. For a low sampling ratio of 15%, we get a reasonable target reconstruction for complex scene using DeepGhost. However, to achieve better results on overall dataset and diverse scenes, we resort to ***S*** = 0.2–0.25 for practical imaging. At such low sampling rates, both *DGI* and Sparse (*DCT* *based*) algorithms fail to reconstruct a clear target.

### Comparison with deep learning algorithms

Furthermore, we design an experiment to validate the superior performance of our deep learning network by comparing it with two existing deep learning networks used for CGI under similar settings. Specifically, we train the models of^16^ (GIDL) and^17^ (DLGI) along with DeepGhost on STL-10 dataset at a low sampling ratio of 0.2. For all three networks, we use similar network parameters (weights, strides, initializations, activations, learning rate etc.).

### Results and analysis

**Figure 4:**
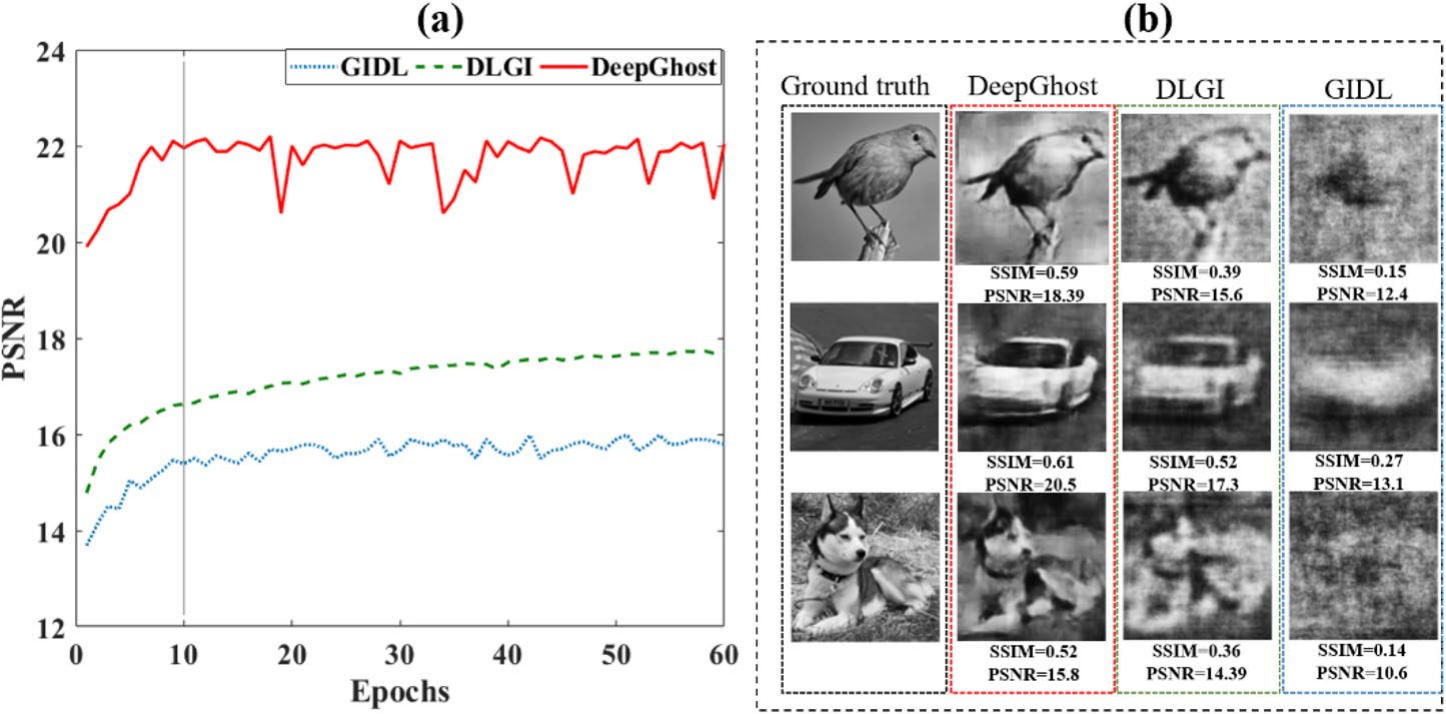
Performance comparison (for GIDL, DLGI, and DeepGhost) (**a**) on test set during training, (**b**) qualitative and quantitative comparison of reconstructions.

The PSNR over the test set (1,000 images) is computed during training and plotted against training epochs, shown in Fig. 4a. The PSNR for the reconstructed image is calculated with respect to its ground truth counterpart. It can be seen from Fig. 4a that it is very challenging for the GIDL network to recover image details from an under sampled image, achieving low PSNR values throughout its training. This is easy to understand because fully-connected neural networks are not ideal for image analysis. Although they can perform well on simple (e.g., digits) dataset, it is difficult for them to achieve satisfactory performance on complex images. Moreover, the training time for the GIDL network is very long compared to DeepGhost due to its fully connected structure. Compared to GIDL, the DLGI employs a better network based on convolutional layers. However, from Fig. 4a, it can be seen that DeepGhost also outperforms DLGI in terms of image reconstruction quality with high PSNR values achieved within a few epochs.

It is important to highlight that the training convergence for DeepGhost is faster compared to both DLGI and GIDL networks. This points toward the fact that simply using deep networks for image reconstruction may not lead to a satisfactory performance. Since DeepGhost uses skip connections along with deep architecture, it can achieve better results with fast convergence. Keeping in view the long convergence times of other models compared to DeepGhost, we carry out comparison testing at a high learning rate (*lr* = 0.001). It can be seen from Fig. 4a that DeepGhost has a chirpy PSNR response after ~ 10 epochs. This is because our network converges faster at a high learning rate compared to DLGI and GIDL networks and then goes into overfitting mode. Therefore, we choose a lower learning rate (*lr* = 0.0001) for DeepGhost training. To further investigate performance differences between these networks, a qualitative comparison is presented in Fig. 4b.

From Fig. 4b, it can be seen that the GIDL network fails to reconstruct complex targets because of its fully connected architecture. Therefore, this kind of network is not suitable for dynamic CGI. Similarly, the DLGI network, by using shallow convolutional structure, roughly estimates the target, failing to provide a clear reconstruction. In contrast, DeepGhost provides much better reconstructions for complex diverse targets. This superior performance of DeepGhost can be attributed to its denoising autoencoder structure with skip connections, which achieves deep architecture with low computational time. The inclination towards using simple architecture, shallow network (to reduce computational time), and validating model on limited data results in poor performance of DLGI and GIDL.

**Figure 5:**
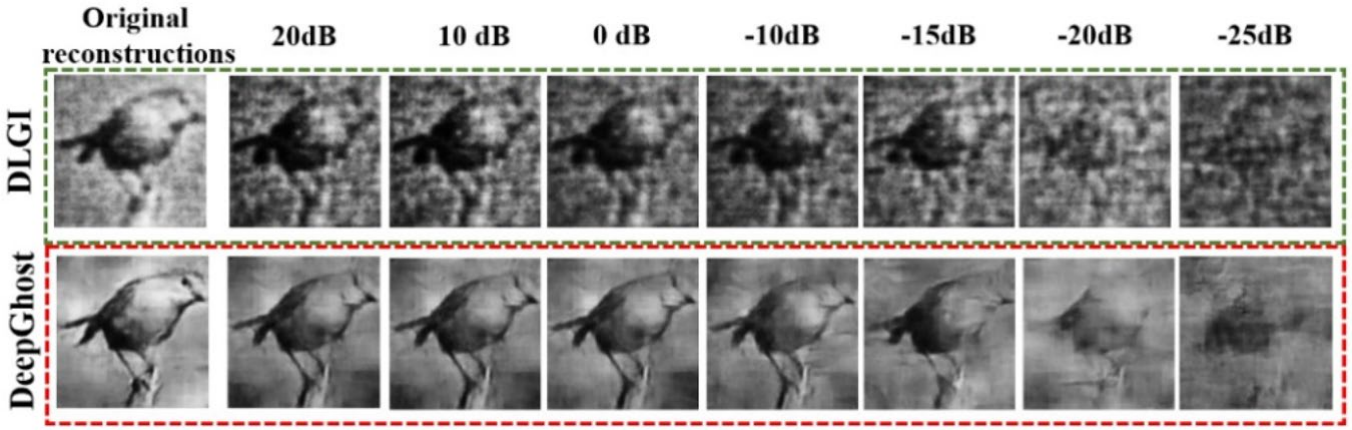
Qualitative comparison of DeepGhost with DLGI for noise robustness (at different noise levels, ***S*** = 0.2).

**Figure 6:**
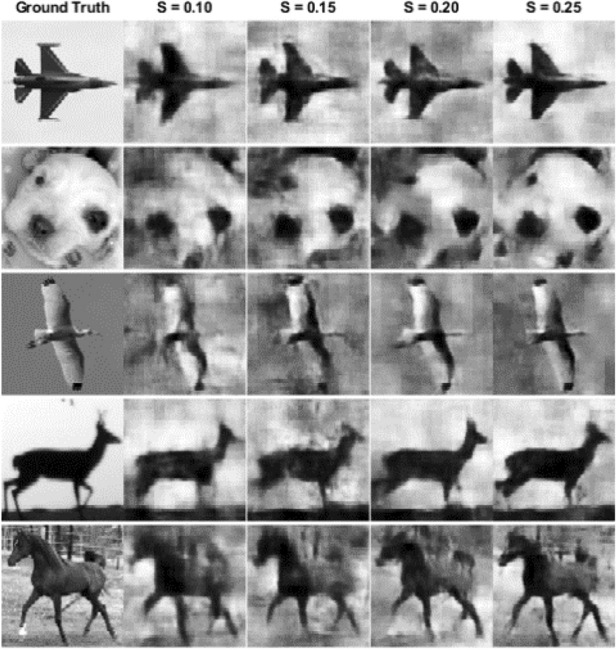
Simulation based image reconstruction using DeepGhost for different sampling ratios.

For evaluating noise robustness, the performance of DeepGhost is compared with DLGI (which gives slightly better reconstruction than GIDL). In this experiment, the detection fluctuations are simulated by adding noise (using *awgn()* function in Matlab) to measurement data (intensity values), resulting in different SNRs. The reconstruction results for the ‘bird’ image at ***S*** = 0.2 are shown in Fig. 5. From qualitative comparison in Fig. 5, it can be seen that the DLGI network fails to combat noise with poor reconstruction quality at different SNRs. This indicates that the convolutional layers (of DLGI) with no mechanism to suppress noise fail to recover a clean target. On the other hand, the DeepGhost network based on denoising autoencoder architecture, learns to suppress noise using compressing/decompressing stages, recovering clean targets at different SNRs. This noise suppression is further aided by skip connections, which provide high frequency information across different layers, to recover fine details which are lost during noise suppression. From overall comparison, it can be concluded that the DeepGhost model is more suitable for practical CGI compared to existing networks. The reconstruction results for DeepGhost at different sampling ratios are shown in Fig. 6.

### Physical experiments

**Figure 7:**
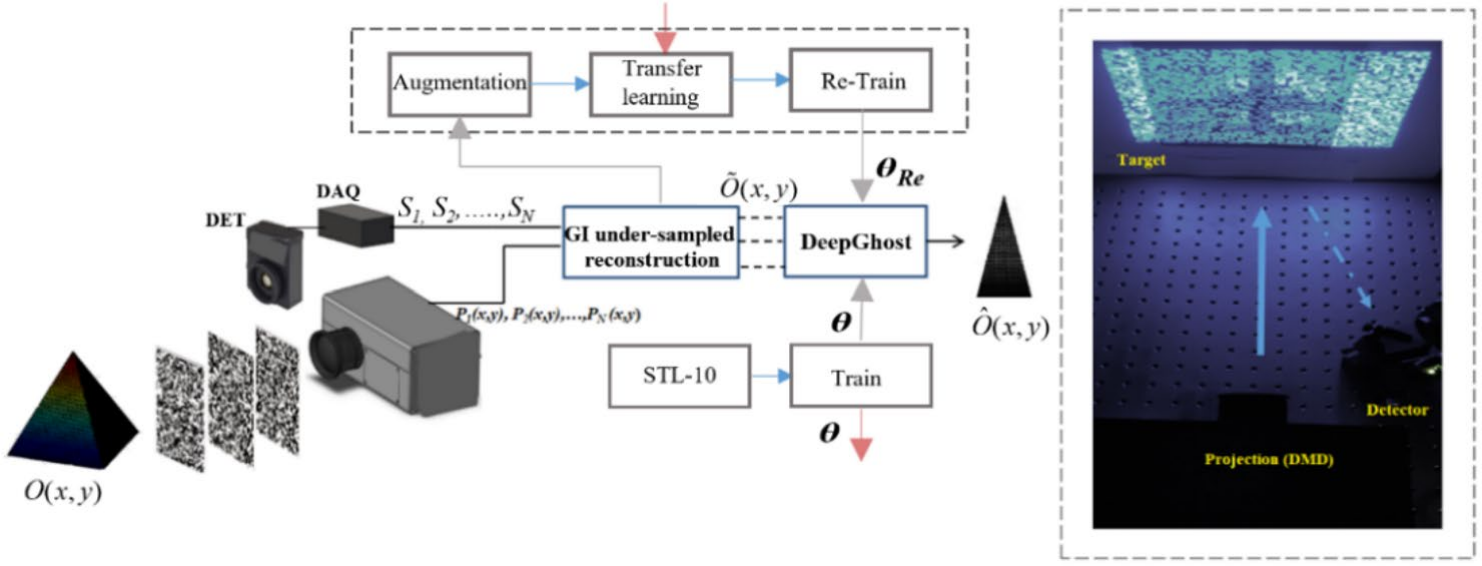
DeepGhost experimental setup.

The experimental arrangement of CGI setup is shown in Fig. 7. A series of random binary patterns is projected using a custom-made projection system. Light from the source LED is modulated by a TI DLP6500 DMD. A projection lens with focusing dial is used to project sharp patterns on the target plane. Target scenes are printed on an A4-sized white paper (using a regular printer). The target is placed at a distance of 500 mm from the plane of projection and detection. Light back-reflected from the scene is collimated on the photodetector (Thorlabs; 21 mm^2^ active area) by a 5 mm imaging lens. Intensity measurements captured by the photodetector are digitized by a 16-bit data acquisition (DAQ) card (Sampling at 2 MS/s). A customized software is used to project patterns and acquire intensity values (using a synchronous trigger) for computation. The rudimentary image reconstructed by the software is passed down to DeepGhost for clean undersampled reconstruction. The data collection and preparation (of experimental and synthetic data) for training takes a week.

#### Experiment-1 results

**Figure 8:**
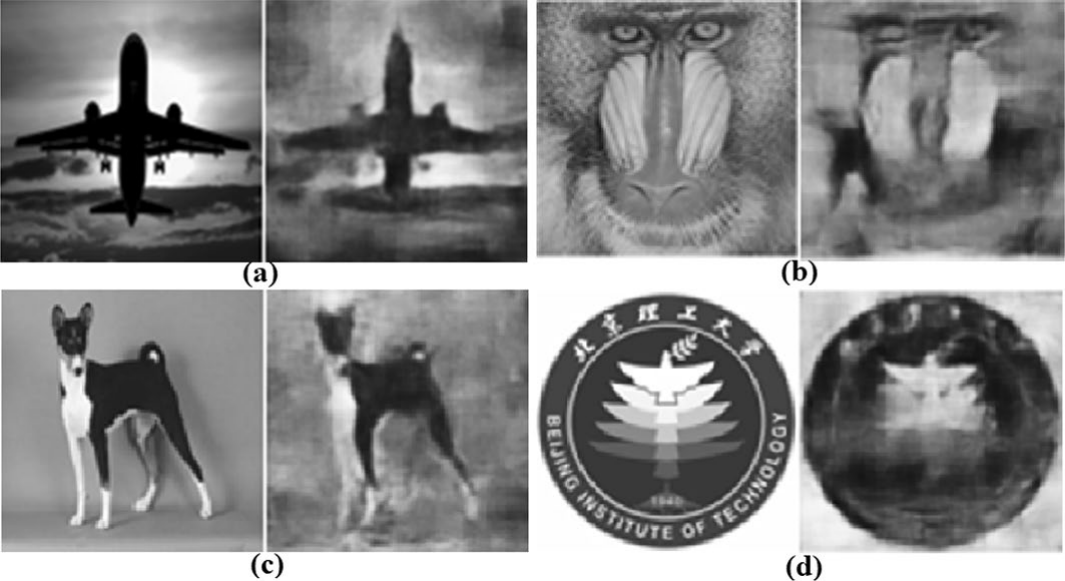
Reconstructions by simulation-trained model on diverse images at ***S = 0.4***. (**a**) SSIM = 0.5521, PSNR = 17.20 dB, (**b**) SSIM = 0.4812, PSNR = 13.22 dB, (**c**) SSIM = 0.6014, PSNR = 19.91 dB, (**d**) SSIM = 0.4613, PSNR = 14.56 dB.

In the first experiment, we directly apply the DeepGhost model trained on simulation dataset to reconstruct target images acquired from random image datasets (airplane and dog image^28^, standard mandrill test image, and our university logo). It is observed that the application of simulation-trained model under physical conditions (e.g., noise, target reflectivity) demands undersampled input to be reconstructed at ***S*** = 0.4. Therefore, we capture input images at 40% sampling rate with respect to clear target reconstruction through our CGI (DGI) setup in this case. Figure 8(a,c: good case, b,d: worst case) shows the reconstructed images with corresponding PSNR and SSIM values. From Fig. 8, it can be seen that the network is able to reconstruct random images from different classes. However, the network is unable to correctly reconstruct all random targets with clarity because of limited data training and knowledge of physical imaging environment. In fact, it is very challenging to optimize a DL model for CGI directly through simulation data for reconstructing diverse random scenes. To counter this problem, we apply augmentation and transfer-learning in our experiments.

#### Experiment-2 results

**Figure 9:**
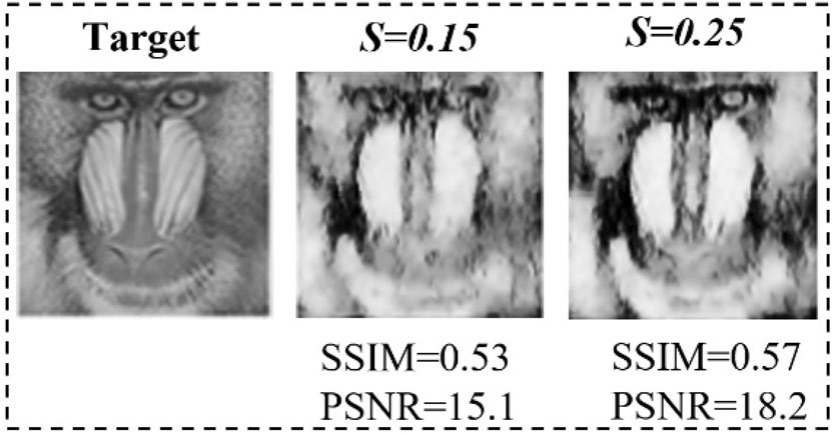
Results for experiment-2 on ‘mandrill’ test set image (as unseen target).

**Figure 10:**
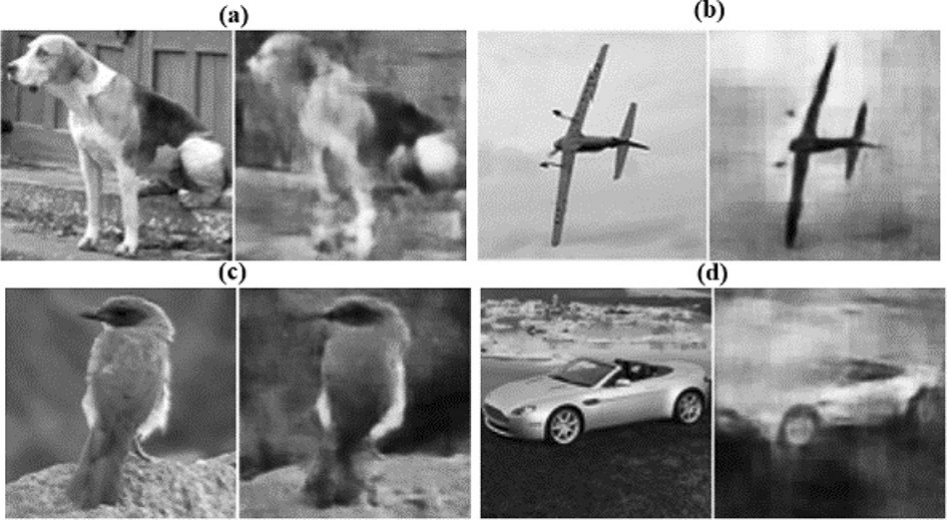
Validation set image reconstruction. (**a**) SSIM = 0.5214, PSNR = 17.62 dB, (**b**) SSIM = 0.6518, PSNR = 18.77 dB, (**c**) SSIM = 0.6913, PSNR = 18.79 dB, (**d**) SSIM = 0.4645, PSNR = 15.12 dB.

In the second experiment, the proposed network is trained on undersampled images acquired from the CGI setup (through DGI for different targets), with ground truth counterparts set as training output. To increase limited data acquired from physical setup, we apply data-augmentation technique (using Keras’s DataGenerator module; by applying translation, rotation, and adding noise in the images). Even though, the data can be increased through augmentation, it is still prone to overfitting. Therefore, we further use transfer-learning to make the network highly-scalable. Transfer-learning is used to provide prior-knowledge from the large dataset (obtained during training) to the smaller augmented dataset to perfect imaging under physical conditions. The results for ‘mandrill’ test image are presented in Fig. 9. It can be seen that the results from experiment-2 (Fig. 9) are very clear compared to the result (Fig. 8b) from simulation based model. The results on validation dataset are understandably consistent, shown in Fig. 10. Overall, it is observed that simple targets with plain background are easily reconstructed at ***S*** = 0.2.

**Figure 11:**
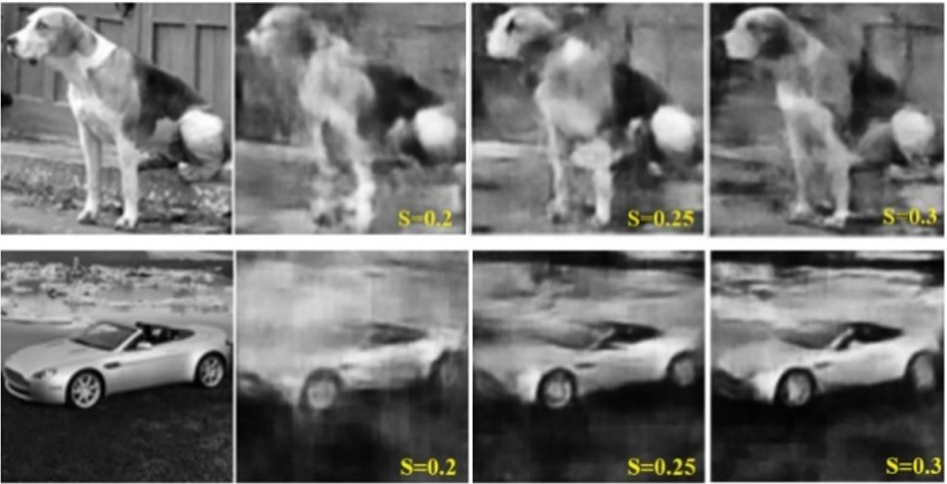
Improving image quality by increasing sampling ratio (***S*** = 0.2, 0.25, and 0.3).

However, for some complex targets (e.g., Fig. 10a,d), better image quality is achieved at a slightly higher sampling ratio (Fig. 11). This is due to (1) practical system noise that can blur reconstructed images by corrupting feature extraction and/or (2) complex image features of random unseen images. The overall results indicate that the reconstruction quality with 20% sampling rate using binary random patterns based CGI is very promising. Although the network can produce better quality reconstructions at higher sampling ratios, it can further be trained on more data to achieve high-quality and reliability at lower sampling rates.

### Imaging time

To quantify imaging time, different values of time for the DeepGhost model are presented in Table 1. The imaging time is based on reconstructing 96 × 96 images at ~ 20 kHz modulation rate. The total imaging time (I_T_) is equal to data acquisition time (I_AQ_) + reconstruction time (I_R_). The reconstruction time (I_R_) is the combined time of DGI (undersampled reconstruction) + DCAN processing. The reconstruction time remains the same for different sampling ratios, which is an attractive feature of DL based model. It can be seen from Table 1 that DeepGhost can achieve real-time frame rates (fps) compared to conventional methods with high reconstruction overhead only.

**Table 1:** Time breakdown for practical imaging.

METHOD	# of projections	I_A_ (s)	I_R_(s)	I_T_ (s)	*fps*
Sparse	–	–	32.61	–	–
TV	–	–	18.63	–	–
DEEPGHOST 15%	1,382	0.069	0.13	0.199	5
DEEPGHOST 20%	1843	0.092	0.13	0.222	4
DEEPGHOST 25%	2,304	0.115	0.13	0.245	4

## Methods

### Principles and methods of CGI

In computational ghost imaging, a target scene *O*(*x,* *y*) is reconstructed by correlating a series of modulation patterns *P*_*i*_(*x,* *y*) with intensity measurements *S*_*i*_ at the bucket detector. The target scene can be reconstructed by^29^:

1 $$ O\left( {x,y} \right) = \left\langle {\left( {S_{i} - \left\langle {S_{i} } \right\rangle } \right)\left( {P_{i} \left( {x,y} \right) - \left\langle {P_{i} \left( {x,y} \right)} \right\rangle } \right)} \right\rangle $$

where *S*_*i*_ is the *i*th measurement, *P*_*i*_is the *i*th modulation pattern, and the ensemble average for *N* iterations is given by: $\left\langle {t_{i} } \right\rangle = \frac{1}{N}\sum\nolimits_{i = 1}^{N} {t_{i} }$. To reconstruct high quality image, a large number of measurements are required.

To improve the performance of correlation based GI, DGI has been proposed^26^. Figure 3 shows images reconstructed using DGI defined by Eq. (2), where, *R*_*i*_ is the reference signal. It is evident that even with these methods, GI still requires a large number of measurements (long imaging time) to produce quality image.

2 $$ O\left( {x,y} \right) = \left\langle {P_{i} \left( {x,y} \right)S_{i} } \right\rangle - \frac{{\left\langle {S_{i} } \right\rangle }}{{\left\langle {R_{i} } \right\rangle }}\left\langle {R_{i} P_{i} \left( {x,y} \right)} \right\rangle $$

To reduce reconstruction time for CGI, compressive sensing methods have been applied to ghost imaging^11^^,^^30,31^. The CS theory allows an object (target scene) *O*(*x,* *y*) to be reconstructed from a set of undersampled measurements *S*, assuming that object is sparse within a fixed basis. For evaluation, we process our GI data with two commonly used priors for natural images: the sparse prior and the total variation (TV) regularization prior. The sparse representation prior^32^ considers natural image to be represented by an orthogonal basis (discrete cosine transform) transform matrix **D** and coefficient vector **c**. The reconstruction for CGI is achieved by minimizing the following function:

3 $$ \mathop {\min }\limits_{O} \left\{ {{\text{f } = \text{ }}\left\| c \right\|_{{l_{1} }} + \frac{{\mu_{1} }}{2}\left\| {DO - c + \frac{{y_{1} }}{{\mu_{1} }}} \right\|_{{l_{2} }}^{2} + \frac{{\mu_{2} }}{2}\left\| {PO - S + \frac{{y_{2} }}{{\mu_{2} }}} \right\|_{{l_{2} }}^{2} } \right\} $$

where *y* is the Lagrange multiplier and *µ* is the balancing parameter. The above *l*1-minimization problem can be solved by using augmented lagrange multiplier (ALM) method^33^. The TV regularization prior is related to the gradient of an image. If **G** is the gradient matrix of an image, the TV regularization prior based reconstruction is given by solving the following minimization:

4 $$ \mathop {\min }\limits_{O} \, \left\{ {{\text{f } = \text{ }}\left\| c \right\|_{{l_{1} }} + \frac{{\mu_{1} }}{2}\left\| {GO - c + \frac{{y_{1} }}{{\mu_{1} }}} \right\|_{{l_{2} }}^{2} + \frac{{\mu_{2} }}{2}\left\| {PO - S + \frac{{y_{2} }}{{\mu_{2} }}} \right\|_{{l_{2} }}^{2} } \right\} $$

### DeepGhost

**Figure 12:**
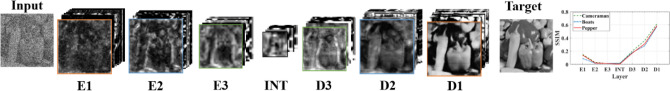
End-to-end Visualization of activation feature maps at different layers in the network. The SSIM plot for different standard test set targets is given to quantify SSIM at different layers. The SSIM increases when the decoding layers start reconstructing.

The proposed deep convolutional autoencoder architecture is shown in Fig. 1. The network employs convolutional layers with trainable filters for extracting features and filtering corruptions from the image. The encoding stages use 32, 64, and 128 (Conv2D) filters for scaling down the data. The compressed data is grouped at an “intermediate” layer with 256 conv-filters. The decoding stages use 128, 64, and 32 filters for reconstructing the encoded image. The output is reconstructed using a single conv-filter at the end. To visualize data processing at each layer, the feature maps for an unseen target (pepper test image) through the network pipeline are shown in Fig. 12. To prevent network operation in saturated or dead regions of activation, the network is initialized with Xavier initialization^34^. After every convolutional layer, batch normalization layer^35^ is used to achieve training efficiency. The data along the pipeline is scaled into different dimensions using max-pooling and Up-sampling operations. To counter data over-fitting, Gaussian noise layers are used to apply regularization through additive Gaussian noise in the hidden layers. The image reconstruction quality is improved by training the network with noisy data traversed via skip connections between similar scale stages. The nonlinearity between layers is created using a nonlinear activation (ReLU).

In general, the autoencoder serves the purpose of image denoising. If O(*x,* *y*) is assumed to be the target, then the target imaged by CGI using undersampled measurements is a corrupted version of the target $g\left( {O\left( {x,y} \right)} \right) + n$ added with noise, represented by $\tilde{O}\left( {x,y} \right)$. The inverse problem of recovering the original image from an undersampled image is solved by applying DL. Through training, the network learns an end-to-end mapping from $\tilde{O}\left( {x,y} \right){\text{ to }}O\left( {x,y} \right)$. For the reconstructed target $\hat{O}\left( {x,y} \right)$, the network is trained on a set S = {*DGI*_undersampled_, *Ground* *truth* }, to minimize the loss function expressed as:

5 $$ \, \ell { (}\theta {) = }\frac{1}{m}\sum\limits_{i = 1}^{m} {\left[ {\hat{O}(x,y) - O(x,y)} \right]}^{2} $$

The network is fed with an undersampled ghost image reconstructed from CGI data using iterative DGI algorithm (Eq. (2). For further time reduction and fast reconstruction, a compressive sensing algorithm can also be used to preprocess CGI data^17^. The network parameters are updated using Adaptive moment estimation optimization^36^ with standard back propagation on mini-batch(es)$$\underset{\raise0.3em\hbox{$\smash{\scriptscriptstyle-}$}}{S}$$ . The learning rate for each layer = 10^–4^. The proposed network is trained on gray-scaled STL-10^25^ 96 × 96 images. All images are preprocessed using standard normalization procedure. The training set has 10,000 images, whereas both test and validation image sets have 1,000 images each. The network is implemented with Keras (TensorFlow support) on an Intel i7 CPU with 32 GB memory.

## Conclusion

In this paper, we demonstrate a DL based imaging framework to improve the performance of random-pattern based CGI. DL can learn features from a large dataset and is more flexible compared to CS optimization techniques based on fixed priors and rigid calculations. The proposed method is capable of reconstructing good-quality 96 × 96 target with 80% compression at 4-5 Hz frame rates. Optimizing random-pattern based CGI for real-time application is very challenging because of its long reconstruction time. Even if the reconstruction time is reduced by means of undersampling, the reconstruction quality of undersampled CGI (through CS or DL) for diverse unseen targets is poor. The main objective in this paper is to reconstruct diverse unseen targets with accuracy. By importing prior knowledge from a large dataset, and training a network on physical data, this objective is achieved. The core component of our imaging framework is the DCAN. The network uses an encoding–decoding architecture combined with skip connections to reconstruct good quality image from an undersampled input. Deep learning combined with GI is a good choice in order to avoid complex methods that fail to reap the benefits of GI i.e., reduced cost and simplicity. By further training our algorithm on a larger dataset (more classes), we can enhance its feature learning ability, which would increase reconstruction reliability and quality. Experimental results show that the proposed method achieves better performance than compressive sensing and existing deep learning methods used for computational ghost imaging.
